# *The Organon* and Materia Medica Club of the Bay Cities of California

**Published:** 1897-03

**Authors:** W. E. Ledyard

**Affiliations:** Secretary


### *THE ORGANON* AND MATERIA MEDICA CLUB OF THE BAY CITIES OF CALIFORNIA.

The regular semi-monthly meeting was held at the office of Dr. George H. Martin, 606 Sutter Street, San Francisco, Friday evening, October 2d, 1896.

The meeting was called to order by the President at 8 P. M.

Members present: Drs. J. M. Selfridge, Augur, Martin, Wilson, and Ledyard.

The minutes of last meeting were read, corrected, and approved.

Sections 190 to 195 of *The Organon* were read and discussed as follows:

Dr. Wilson (referring to Section 190)—A wholesale admonition not always regarded by homœopathic physicians. Are not curetting the womb and packing with iodoform gauze prejudicial to the patient?

Dr. J. M. Selfridge—Most decidedly so. I had a case of eczema in a boy treated unsuccessfully by external applications. It was a *Rhus* case. The case was exceedingly difficult on account of the complicated treatment. He would scratch until the blood would run. *Psorinum*$^{200}$ and CM gave no relief. *Eczema*$^{cm}$ likewise failed. After waiting for quite a while gave *Sulph.*$^{cm}$, *one dose, with marked improvement for six weeks.* The improvement ceased, and after a second dose the improvement is going on again.

Dr. Augur—Referring to the treatment of facial erysipelas, stated that in his old-school days he applied *Collodion* and a 10 per cent. solution of *Iodoform;* latterly, however, he uses no local medications, simply applying linen wrung out of warm water. He cited a case of *phlegmonous erysipelas, extending from right to left,* in which there was great sac-like œdema of the eyelids, and such intense inflammation that the case went on to suppuration with sloughing of the cuticle of the eyelids. The eyes were also much involved, with loss of sight in the right eye. In this case *Apis* "acted like a charm." I have met homœopathic physicians who apply *Collodion* and *Veratrum-album,* but am of the opinion that the latter would interfere with the curative action of the remedy.

Dr. Ledyard suggested that where Apis is indicated there is *relief from cold applications.* Erysipelas, commencing on *right side and going to left,* calls for *Graphites,* while in the case of *gangrenous erysipelas* Ars., Carb-v., Lach., Rhus or Secale is likely to be of service. He referred to a case of erysipelas of the face and scalp which had come to his knowledge, in which, after local treatment, *the patient died, having previously gone into a stupor;* this fact evidently pointing to *metastasis to the brain,* which might even then have been cured by Lach., which meets this indication.

Dr. Wilson is in favor of *dry applications,* especially cotton batting.

Dr. Augur interrogated the Club with regard to its opinion of *Collodion* locally.

Some of the members saw no objection to its use.

Dr. J. M. Selfridge never applies anything but linen wet with tepid water.

Dr. Martin sets much store by *corn-starch, dredging it on from a spice-box.* He has found it very soothing.

Dr. Wilson put the question as to what the old school considers "isolated."

Dr. J. M. Selfridge remarked that old-school physicians have frequently asserted that cancer in its incipiency is purely local and can then be cured by the knife.

They look upon a tumor or other local manifestation as a parasite.

Dr. Augur, being appealed to as the latest authority from the old school, said he could not conceive of any part of the body as isolated, even when practicing as an old-school physician.

Dr. Ledyard stated that in the London hospitals *chancres were invariably cauterized,* sometimes with even fuming Nitric-acid.

Dr. Martin put the query with regard to the number of physicians who seek to investigate all the *local* symptoms, while they overlook the others.

Dr. Ledyard replied for himself, that he invariably investigated *all the symptoms, especially the mental.*

Dr. Augur stated that in "taking a case," you commence to question, in true Hanemannian style, about the head, stomach, bowels, and other parts of the body, when the patient, somewhat *impatiently,* ejaculates: "Oh, but, Doctor, I *don't care about anything but the stomach ;* that is the only thing I came to you to have rectified."

Dr. Ledyard—I have had one case of *chancre cured by one dose of Mercurius high,* and several cases cured by a few doses of the same medicine.

Dr. Martin, referring to the middle of Section 194, said: We are liable to forget that when patients are on the road to recovery, especially in cases of stomach trouble, that they should continue under treatment. Regulating the habits of a patient is not sufficient.

Dr. Wilson remarked concerning the wisdom of keeping patients under treatment until the cure is completed.

Motion to adjourn and meet the third Friday of the month at 606 Sutter Street was adopted.

W. E. LEDYARD, B. A., M. B., etc.,
*Secretary.*

